# Recursive Path Model for Health Literacy: The Effect of Social Support and Geographical Residence

**DOI:** 10.3389/fpubh.2021.724995

**Published:** 2021-09-28

**Authors:** Éva Bíró, Ferenc Vincze, Gabriella Mátyás, Karolina Kósa

**Affiliations:** ^1^Department of Public Health and Epidemiology, Faculty of Medicine, University of Debrecen, Debrecen, Hungary; ^2^Doctoral School of Health Sciences, University of Debrecen, Debrecen, Hungary; ^3^Department of Behavioral Sciences, Faculty of Medicine, University of Debrecen, Debrecen, Hungary

**Keywords:** health literacy, social support, socioeconomic position, permanent residence, recursive path model

## Abstract

**Background:** The public health relevance of health literacy is highlighted by the fact that its higher levels can improve health outcomes and reduce health inequities. In order to design effective interventions for improving health literacy, the relationship between health literacy and other factors such as sociodemographic variables, subjective health and social support must be understood.

**Objective:** Our aim was to test a socioecological model of the determinants of health literacy with a special focus on the effect of residence. Our study investigated geographical differences regarding the levels of health literacy and its determinants as this was not investigated before in European nationwide surveys.

**Methods:** Data was collected by a polling company in a sample (*n* = 1,200) of the Hungarian adult population nationally representative by age, gender, and permanent residence in 2019 January. The questionnaire included items on sociodemographic data, subjective well-being, social support, and two health literacy scales. A recursive path model was used to outline the mediating effect of social support between sociodemographic variables and health literacy where both direct and indirect effects of the explanatory variables and multiple relationships among the variables were analyzed simultaneously. Multiple-group analysis was applied to the three pre-set categories of permanent residence (capital city, urban and rural).

**Results:** There was no statistically significant difference by residence regarding levels of health literacy. Social support and educational attainment were the most important determinants of health literacy after adjusting for the effect of other sociodemographic variables. However, the magnitude of effect of social support and educational attainment is different between types of settlements, the strongest being in rural areas.

**Conclusion:** Social support seems to mediate the effect of socioeconomic position on health literacy which could be taken into account when designing interventions to improve health literacy, especially in rural areas. Further studies would be needed especially in rural communities to see whether improvement of social support could be utilized in projects to increase the level of health literacy.

## Introduction

An increasing number of articles reflects a growing scientific interest in health literacy (HL). According to one of the leading expert groups in the field, HL is “linked to literacy and entails people's knowledge, motivation and competences to access, understand, appraise, and apply health information in order to make judgments and take decisions in every day life concerning healthcare, disease prevention and health promotion to maintain or improve quality of life during the life course.” (1) A plethora of health literacy measures exist that can be grouped into two main categories: self-report (subjective) measures and performance-based (objective) tools (2). The level of HL is often dependent on the used measurement therefore it is important that researchers choose one which is aligned with the research question and has been validated in a similar target population. The public health relevance of health literacy is highlighted by the fact that its higher levels can improve health outcomes and reduce health inequities (3). In order to design effective interventions for improving HL, the relationship between HL and other factors such as sociodemographic variables, subjective health and social support must be understood.

According to the conceptual framework of the World Health Organization's Commission on Social Determinants of Health socioeconomic position (SEP) has a main impact on equity in health (4). The most commonly used proxy indicators of SEP include income, education, occupation and gender. SEP has a major role in generating health inequities. Low SEP is associated with low level of HL of which education is the most important determinant. HL seems to be a mediating factor between SEP and health-related outcomes such as health status, quality of life, health behavior, and use of preventive services (5–7). If HL is a mediator between SEP and health status, it is potentially modifiable, and its improvement at the individual and population level can reduce health disparities (6).

Differences in levels of HL between rural and urban populations was assessed by a recent systematic review which found that urban populations tend to have higher levels of HL than rural ones. Rurality itself does not explain differences in HL, but SEP may play a role in it. This potentially can be explained by the fact that rurality in some cases can be treated as a proxy of low SEP depending on its definition (8).

There are studies suggesting that the correlation between SEP and health is partly genetically confounded (9–11). A recent twin cohort study revealed that both genetic and environmental factors can influence individual differences in educational attainment, though the effect of genetic factors seems to have decreased (12). However, a public health perspective requires focusing on determinants that are potentially modifiable at the population level. In line with the position of the World Health Organization (4), namely that socio-economic position is dominantly determined by non-biological (social, economic, political) factors, our study aimed at uncovering the relation of such non-biological factors.

From the other side there is growing evidence that there is a need for greater inclusion of social cohesion (social capital, social support) in health literacy research. Based on previous results it seems that social cohesion plays an important role in HL, but the exact mechanism is still unknown (13).

HL was measured by two surveys in the Hungarian general population in 2015. One of them was implemented in one county in a sample of 302 people that was produced in two waves. First, convenience sampling was carried out followed by sampling to produce a sample representative by gender, age, and education (14, 15). This survey aimed at validating the Hungarian version of the Short-Test of Functional Health Literacy in Adults (S-TOFHLA) questionnaire and the Chew screening questions (16, 17). Results of this countywide survey showed that 86% of the participants had adequate level of HL measured by the S-TOFHLA questionnaire. Significant correlation between SEP (education level and income) and HL was found (*p* < 0.001). A nationwide survey conducted by Koltai and Kun measured objective and subjective HL in a representative sample of 1,008 people (18, 19) using the European Health Literacy Survey Questionnaire 47 (HLS-EU-Q47) (20) and the Newest Vital Sign (NVS) tool (21). According to their results, 68% of the participants had adequate levels of objective HL measured by NVS (18). This is a particularly good result in European comparison considering that only the Netherlands had better result with 76% of the population at adequate levels of objective HL in the European Health Literacy Survey (HLS-EU) covering 8 countries. Overall, 55% of the European participants had an adequate level of objective HL in the HLS-EU survey (20). On the other hand, Hungarian results in terms of subjective HL measured by the HLS-EU-Q47 were unfavorable with 52% of the sample falling into the insufficient or problematic category compared to the European average of 47% (19).

Yet another pilot project (22) measured SEP, health status, knowledge about triage system and HL using the HLS-EU-Q47 (20) in one county (Baranya) of Hungary in 2019 with 141 respondents. Nearly half of the participants (46.1%) had limited HL levels. Significant correlation between the level of HL and education (*p* = 0.02), training in a healthcare profession (*p* = 0.001) and economic status (*p* = 0.035) were found. Significant difference in HL was found between those with low and high educational level (*p* = 0.018). In addition, a difference between the levels of HL in rural and urban population was revealed. Rural people were found to have a lower level of HL compared to people living in urban areas (*p* = 0.043), but in that analysis, the impact of SEP was not controlled.

Our aims were (1) to investigate the hypothetical relationship between SEP and health literacy—measured simultaneal from a subjective and objective point of view—controlled for geographical residence and the mediation effect of social support; (2) to uncover geographical differences in the level of health literacy and its determinants as this was not investigated in European nationwide surveys before.

## Materials and Methods

### Study Population and Data Collection

Data was collected by a polling company in a sample of the Hungarian adult population nationally representative by age, gender, and permanent residence in 2019 January.

The sample consisted of 1,200 persons aged 18 years or older. Four-stage random sampling was used in which 120 sampling points were selected proportionally by settlement size, then the starting points of the interviewers in each sampling unit were randomly selected. Ten households in each sampling unit were reached by a random route method, and one respondent was selected in each household by the Kish selection grid (23).

The paper-based questionnaire was administered by an interviewer. All interviewees were informed about the voluntary nature of participation and its conformation to the requirements of the national data protection act; none of them received incentive in any form. The company follows the professional and ethical guidelines specified in the ESOMAR Code of Conduct (24). Informed consent was obtained during data collection, and the appropriate ethical standards (according to the World Medical Association's Declaration of Helsinki) were followed as acknowledged by the Medical Research Council of the University of Debrecen (5315–2019).

### Domains of the Questionnaire

The questionnaire included items on demographic and socioeconomic data, subjective well-being, social support, and two newly adapted scales in order to measure subjective (Brief Health Literacy Screening Tool, BRIEF) and objective (NVS) health literacy. Items not referred separately were taken from the tool of the Hungarian version of the European Health Interview Survey of 2014 (25).

#### Demographic and Socioeconomic Data

Age, gender, marital status (unmarried, married, divorced, widowed), type of the settlement of permanent residence (capital city, urban/city, rural/village), education (primary school or less, vocational, high school, college/university), employment status (active, inactive, retired, student; during the analysis these were dichotomized as active or student and inactive or retired), and subjective perception of family wealth (very bad, bad, average, good, very good) were registered.

#### Self-Perceived Health

Perceived health was measured by a standard question by respondents assessing their health on a five-point Likert scale from very bad to very good.

#### Social Support

Perceived social support was measured by the Oslo Social Support Scale from the European Health Interview Survey 2014. The scale contains three questions inquiring about the number of people the respondents can rely on in difficult life situations, how much concern other people show in what respondents are doing, and how easy it is for them to get practical help from neighbors. The sum score for these three items ranges between 3 and 14 with higher score indicating stronger support.

#### Health Literacy

Health literacy was measured by a self-perceived (BRIEF) and an objective measure (NVS). The validated Hungarian versions of both scales were used (26). The NVS satisfied the criteria for internal consistency (Cronbach α = 0.72), while BRIEF questionnaire exhibited very good internal consistency (Cronbach α = 0.87) (26). Higher total scores reflect better health literacy at both scales (21, 27). The sum score for BRIEF ranges between 4 and 20, while this range is 0 to 6 for NVS.

### Data Analysis

Only participants who provided information for all items were included in the analyses. Descriptive statistics were used to describe the respondents' sociodemographic characteristics. Equality of variances of the variables as well as possible outliers were checked before testing. The chi-square (χ^2^) test was used for categorical variables and the Kruskal–Wallis test for continuous variables (with Bonferroni correction for multiple tests) as appropriate.

**Figure 1:**
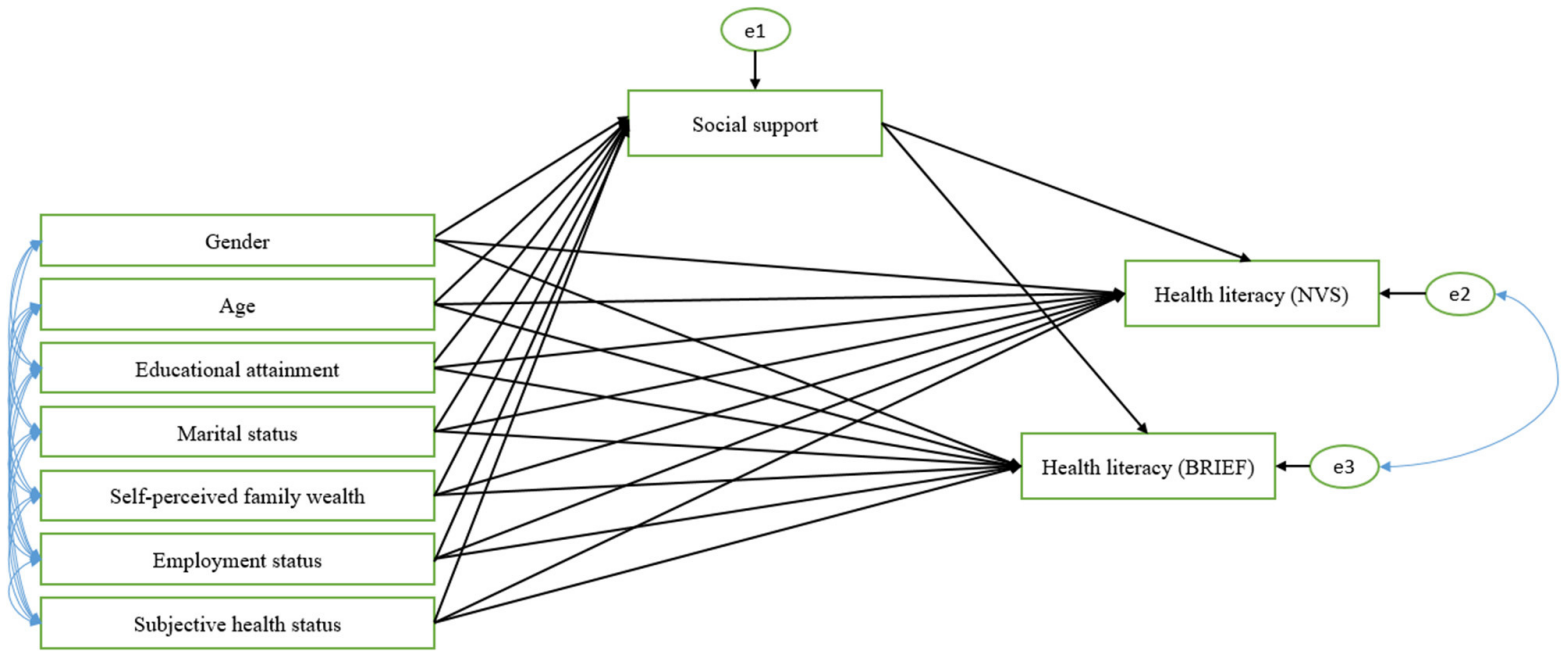
The hypothesized multigroup recursive path model of demographic and socioeconomic factors on social support and health literacy. NVS, Newest Vital Sign; Brief, Brief Health Literacy Screening Tool; Multiple-group analysis was applied simultaneously to the three geographical categories of permanent residence (capital city, urban and rural).

A recursive path model was built to outline the hypothetical relationship between SEP and health literacy controlled for geographical residence and the mediating effect of social support in accordance with the first aim of our study. Model specification was performed based on preliminary hypothesis, model fit and modification indices. Both direct and indirect effects of the explanatory variables and multiple relationships among the variables were analyzed simultaneously (full sample model, Figure 1). Assessment of model fit was based on multiple indicators such as the chi-square statistic (χ^2^), comparative fit index (CFI), goodness-of-fit index (GFI), root-mean-square error of approximation (RMSEA), and p of close fit (PCLOSE). The model fit was considered good in case of non-significant (*p* > 0.05) chi-square statistic, CFI >0.95, and GFI above 0.95. RMSEA <0.05 demonstrates a “close fit” to the data, while *p* > 0.05 for the PCLOSE test indicated that the model has a good fit to the data (28, 29).

Structural relationships of the path model were evaluated using direct (β_d_) and social support mediated indirect (β_i_) standardized path coefficients with the corresponding 95% confidence intervals (95% CI). Indirect effect (social support mediated effect) was analyzed only if all direct effects were significant. Equality of variances of the variables as well as possible outliers and multivariate normality according to Mahalanobis distances were checked before testing. Considering the multivariate non-normality, a bias-corrected (percentile method) bootstrapping procedure (1,000 bootstraps) was used to estimate model parameters.

Regarding the second aim of our study, a multiple-group analysis was applied to the three geographical categories of permanent residence (capital city, urban and rural). While testing for configural invariance, we focused on the extent to which path coefficients of the hypothesized model were similar across respondent's permanent residence. Analysis of the group invariance for the hypothesized model (CM: configural model) was performed by a method constraining two nested models (Model 1 in which all path coefficients were constrained equal, Model 2 where social support and education-related path coefficients were constrained equal) to test sequentially for the equivalence of structural weights. Invariance was tested using the χ^2^ statistical difference (Δχ^2^) and the difference in CFI (ΔCFI). Invariance across groups was satisfied if the Δχ^2^ value between models was not significant and if the ΔCFI overstep the 0.01 threshold (30). Data were analyzed using SPSS 22.0 (IBM Corp, Armonk, NY, USA) and Amos (Version 26.0).

## Results

### Main Characteristics of the Sample

1,200 respondents participated in the study. 93 respondents were excluded in the preliminary analysis due to missing data, providing a database of 1,107 records. Almost two-third of the respondents were female (61%), ~16% had primary school or less as the highest level of education, while the frequency of vocational or high school-educated participants was equal (36–36%). More than half of the respondents were married. Regarding self-perceived family wealth, 20, 56, and 24% of the participants characterized their status as bad, average, and good. Bad subjective health status was observed in 12% of the subjects, and ~56% of the respondents belonged to the active employment status category, or studied in an educational institute. The mean age of subjects was 53.62 (standard deviation, SD: ± 15.91) years. The mean score of NVS was 3.44 (SD: ± 1.88), 14.25 (SD: ± 3.83) for BRIEF, and 10.02 (SD: ± 1.68) for social support (Table 1).

**Table 1:** Characteristics of the study population by place of residence.

	**Residence**			***p*** ^*^	**Total sample (*****N*** **= 1107)**
	**Capital city (*****n*** **= 210)**	**Urban (*****n*** **= 584)**	**Rural (*****n*** **= 313)**		
**Gender**					
Male	76 (36.19%)	233 (39.90%)	121 (38.66%)	0.638	430 (38.84%)
Female	134 (63.81%)	351 (60.10%)	192 (61.34%)		677 (61.16%)
**Educational attainment**					
Primary school or less	16 (7.62%)	75 (12.84%)	83 (26.52%)	**<0.001**	174 (15.72%)
Vocational school	42 (20.00%)	238 (40.75%)	115 (36.74%)		395 (35.68%)
High school	102 (48.57%)	208 (35.62%)	89 (28.43%)		399 (36.04%)
University/college	50 (23.81%)	63 (10.79%)	26 (8.31%)		139 (12.56%)
**Self-perceived family wealth**					
Bad/very bad	37 (17.62%)	108 (18.49%)	78 (24.92%)	**0.003**	223 (20.14%)
Average	122 (58.10%)	315 (53.94%)	183 (58.47%)		620 (56.01%)
Good/very good	51 (24.29%)	161 (27.57%)	52 (16.61%)		264 (23.85%)
**Marital status**					
Unmarried	35 (16.67%)	68 (11.64%)	41 (13.10%)	**0.034**	144 (13.01%)
Divorced	47 (22.38%)	95 (16.27%)	47 (15.02%)		189 (17.07%)
Widowed	34 (16.19%)	81 (13.87%)	51 (16.29%)		166 (15.00%)
Married	94 (44.76%)	340 (58.22%)	174 (55.59%)		608 (54.92%)
**Subjective health status**					
Bad/very bad	21 (10.00%)	61 (10.45%)	51 (16.29%)	0.051	133 (12.01%)
Fair	81 (38.57%)	189 (32.36%)	110 (35.14%)		380 (34.33%)
Good	89 (42.38%)	281 (48.12%)	132 (42.17%)		502 (45.35%)
Very good	19 (9.05%)	53 (9.08%)	20 (6.39%)		92 (8.31%)
**Employment status**					
Active or student	108 (51.43%)	336 (57.53%)	171 (54.63%)	0.289	615 (55.56%)
Inactive or retired	102 (48.57%)	248 (42.47%)	142 (45.37%)		492 (44.44%)
**Age; mean (±SD)**	56.84 (±16.89)	53.08 (±15.32)	52.47 (±16.08)	**0.006** ^**^	53.62 (±15.91)
**NVS; mean (±SD)**	3.30 (±1.74)	3.41 (±1.89)	3.60 (±1.93)	0.135	3.44 (±1.88)
**BRIEF; mean (±SD)**	14.02 (±3.48)	14.36 (±3.86)	14.21 (±3.99)	0.314	14.25 (±3.83)
**Social support; mean (±SD)**	9.68 (±1.67)	10.04 (±1.60)	10.22 (±1.80)	**0.001** ^***^	10.02 (±1.68)

*SD, standard deviation, NVS, Newest Vital Sign, BRIEF, Brief Health Literacy Screening Tool*.

* *Chi-square for ratio associations, Kruskal–Wallis test for mean differences of independent-samples*.

** *Pairwise comparisons of residence with Bonferroni correction for multiple tests: p = 0.017 (Capital city–Urban); p = 0.008 (Capital city–Rural); p = 0.999 (Urban–Rural)*.

*** *Pairwise comparisons of residence with Bonferroni correction for multiple tests: p = 0.052 (Capital city–Urban); p = 0.001 (Capital city–Rural); p = 0.168 (Urban–Rural). Significant differences are marked in bold*.

Significant differences were found for educational attainment, self-perceived family wealth, marital status, age, and social support by permanent residence. However, there was no statistically significant difference by residence among categories of gender, subjective health status, employment status, or the means of NVS and BRIEF (Table 1).

### Analysis of the Recursive Path Model

The fit indices for the structural path model of the entire sample hypothesizing social support as the mediator of sociodemographic effects on health literacy indicated that data fit the model well: the χ^2^ statistics and PCLOSE test were non-significant. The RMSEA (0.026), GFI (0.997) and CFI (0.996) were below their respective thresholds confirming the appropriateness of the model for our data. (A correlation matrix between all variables can be found in the Supplementary Material).

The full sample model indicated that education [β_d_ = 0.10; (95%CI = 0.03; 0.16)], marital status (widowed) [β_d_ = −0.07; (95%CI = −0.14;−0.008)], subjective health [β_d_ = 0.08; (95%CI = 0.01; 0.16)] and social support [β_d_ = 0.11; (95%CI = 0.04; 0.17)] exerted a significant standardized direct effect on NVS. The standardized path coefficients between education [β_d_ = 0.13; (95%CI = 0.07; 0.20)], self-perceived family wealth [β_d_ = 0.11; (95%CI = 0.05; 0.17)], social support [β_d_ = 0.10; (95%CI = 0.05; 0.16)] and BRIEF were also significant (Table 2). Social support mediates the effect of self-perceived family wealth [β_i_ = 0.01; (95%CI = 0.01; 0.02)] and subjective health [β_i_ = 0.03; (95%CI = 0.01; 0.05)] on NVS. The full sample model also indicated an indirect link between self-perceived family wealth [β_i_ = 0.01; (95%CI = 0.01; 0.02)], subjective health [β_i_ = 0.02; (95%CI = 0.01; 0.04)] and BRIEF mediated by social support.

**Table 2:** Full sample: Estimated direct effects of demographic and socioeconomic factors on social support and health literacy as measured by the NVS and BRIEF questionnaires.

	Social support^*^	NVS^*^	BRIEF^*^
Male/Female	0.02 [−0.04; 0.08]	0.01 [−0.05; 0.07]	0.05 [−0.01; 0.11]
Age	0.05 [−0.03; 0.15]	0.01 [−0.08; 0.10]	0.02 [−0.06; 0.10]
Education	−0.01 [−0.07; 0.05]	**0.10 [0.03; 0.16]**	**0.13 [0.07; 0.20]**
Self-perceived family wealth	**0.11 [0.05; 0.18]**	0.004 [−0.07; 0.07]	**0.11 [0.05; 0.17]**
Inactive or retired/Active or student	0.01 [−0.07; 0.08]	−0.03 [−0.11; 0.05]	−0.06 [-0.14; 0.02]
Unmarried/Married	−0.04 [−0.10; 0.02]	−0.01 [−0.07; 0.06]	−0.03 [−0.10; 0.03]
Divorced/Married	−0.04 [−0.10; 0.01]	−0.03 [−0.09; 0.03]	−0.05 [−0.11; 0.02]
Widowed/Married	−0.04 [−0.12; 0.02]	**−0.07 [−0.14;−0.008]**	−0.03 [-0.10; 0.04]
Subjective health	**0.24 [0.16; 0.31]**	**0.08 [0.01; 0.16]**	0.06 [-0.01; 0.14]
Social support	–	**0.11 [0.04; 0.17]**	**0.10 [0.05; 0.16]**

*Overall fit statistics of the model: χ^2^ (df) = 17.650 (10); χ^2^(p–value) = 0.061; CFI = 0.996; RMSEA = 0.026; PCLOSE = 0.978; GFI = 0.997. Significant associations are marked in bold*.

* *β_d_. [95%CI]: β_d_: standardized direct path coefficients; [95%CI]: 95% confidence interval obtained by bias-corrected percentile method of bootstrapping. NVS, Newest Vital Sign, BRIEF, Brief Health Literacy Screening Tool*.

Table 3 presents the results of multiple-group path analysis across the type of residence of the respondents. All residential groups were analyzed simultaneously in the configural model to obtain efficient estimates where all path coefficients were freely estimated. In the subgroup of “capital city,” education was positively [β_d_ = 0.23; (95%CI = 0.11; 0.38)], widowed marital status was negatively [β_d_ = −0.14; 95%CI = (-0.28;−0.002)] related to NVS. The standardized direct effect of education [β_d_ = 0.18; (0.04; 0.31)], self-perceived family wealth [β_d_ = 0.14; 95%CI = (0.004; 0.26)] and social support [β_d_ = 0.19; 95%CI = (0.06; 0.31)] predicted the level of BRIEF. The social support-mediated standardized effect of gender and subjective health was [β_i_ = −0.03; (95%CI = −0.07;−0.01)] and [β_i_ = 0.08; (95%CI = 0.03; 0.15)] on BRIEF, respectively.

**Table 3:** Groups by geographical residence: Estimated direct effects of demographic and socioeconomic factors on social support and health literacy as measured by the NVS and BRIEF questionnaire.

		Social support^*^	NVS^*^	BRIEF^*^
Capital city	Male/Female	**−0.16 [−0.29;−0.01]**	0.05 [−0.04; 0.13]	0.08 [−0.04; 0.19]
	Age	0.22 [−0.04; 0.42]	−0.05 [−0.32; 0.22]	−0.01 [−0.24; 0.20]
	Education	−0.01 [−0.14; 0.12]	**0.23 [0.11; 0.38]**	**0.18 [0.04; 0.31]**
	Self-perceived family wealth	0.12 [−0.01; 0.25]	−0.03 [−0.18; 0.11]	**0.14 [0.004; 0.26]**
	Inactive or retired/Active or student	−0.01 [−0.21; 0.21]	0.05 [−0.19; 0.28]	0.06 [−0.16; 0.27]
	Unmarried/Married	0.04 [−0.11; 0.18]	0.00 [−0.17; 0.16]	−0.02 [−0.18; 0.12]
	Divorced/Married	0.03 [−0.11; 0.18]	−0.06 [−0.24; 0.07]	0.04 [−0.10; 0.18]
	Widowed/Married	−0.04 [−0.19; 0.11]	**−0.14 [−0.28;−0.002]**	−0.14 [-0.27; 0.03]
	Subjective health	**0.40 [0.23; 0.55]**	0.05 [−0.12; 0.22]	0.01 [−0.15; 0.18]
	Social support	–	−0.04 [-0.19; 0.11]	**0.19 [0.06; 0.31]**
Urban	Male/Female	0.05 [−0.04; 0.13]	0.03 [−0.05; 0.12]	**0.11 [0.01; 0.19]**
	Age	0.13 [0.00; 0.23]	0.01 [−0.11; 0.13]	0.03 [-0.09; 0.14]
	Education	0.02 [−0.06; 0.10]	0.06 [−0.03; 0.14]	**0.09 [0.01; 0.18]**
	Self-perceived family wealth	**0.10 [0.01; 0.18]**	0.02 [−0.07; 0.11]	**0.12 [0.04; 0.20]**
	Inactive or retired/Active or student	−0.05 [−0.13; 0.05]	−0.01 [−0.12; 0.09]	−0.04 [−0.14; 0.06]
	Unmarried/Married	−0.02 [−0.10; 0.07]	0.07 [−0.02; 0.16]	0.01 [−0.09; 0.11]
	Divorced/Married	−0.05 [−0.13; 0.04]	0.00 [−0.08; 0.08]	−0.04 [−0.12; 0.05]
	Widowed/Married	−0.02 [−0.12; 0.08]	−0.04 [-0.15; 0.05]	0.00 [−0.10; 0.10]
	Subjective health	**0.24 [0.13; 0.34]**	**0.13 [0.03; 0.22]**	0.06 [-0.06; 0.16]
	Social support	–	**0.09 [0.01; 0.18]**	−0.01 [−0.10; 0.08]
Rural	Male/Female	0.08 [−0.04; 0.19]	−0.04 [−0.16; 0.07]	0.00 [−0.11; 0.11]
	Age	−0.05 [−0.22; 0.12]	0.11 [−0.04; 0.25]	0.06 [−0.09; 0.21]
	Education	0.03 [−0.08; 0.14]	**0.12 [0.02; 0.24]**	**0.17 [0.07; 0.27]**
	Self-perceived family wealth	**0.14 [0.02; 0.25]**	−0.04 [−0.15; 0.08]	0.05 [-0.06; 0.17]
	Inactive or retired/Active or student	0.09 [−0.05; 0.25]	−0.12 [−0.25; 0.02]	**−0.18 [−0.31;−0.03]**
	Unmarried/Married	−0.08 [−0.21; 0.05]	**-0.13 [-0.25;−0.01]**	−0.08 [-0.21; 0.03]
	Divorced/Married	−0.04 [−0.15; 0.08]	−0.07 [−0.18; 0.05]	**−0.13 [−0.24;−0.02]**
	Widowed/Married	−0.10 [−0.21; 0.04]	−0.08 [-0.22; 0.06]	−0.01 [−0.16; 0.10]
	Subjective health	**0.19 [0.05; 0.33]**	0.08 [−0.05; 0.21]	0.13 [0.00; 0.27]
	Social support	–	**0.19 [0.08; 0.30]**	**0.21 [0.10; 0.30]**

*Overall fit statistics of the model, χ2 (df) = 38.420 (30); χ^2^(p–value) = 0.139; CFI = 0.996; RMSEA = 0.016; PCLOSE = 1.000; GFI = 0.994. Significant associations are marked in bold*.

* *β_d_.[95%CI]: standardized direct path coefficients; [95%CI]: 95% confidence interval obtained by bias-corrected percentile method of bootstrapping. NVS, Newest Vital Sign, BRIEF, Brief Health Literacy Screening Tool*.

In the “urban” subgroup, better subjective health [β_d_ = 0.13; (95%CI = 0.03; 0.22)] and higher social support [β_d_ = 0.09; (95%CI = 0.01; 0.18)] predicted higher NVS. Gender [β_d_ = 0.11; (95%CI = 0.01; 0.19)], education [β_d_ = 0.09; (95%CI = 0.01; 0.18)], and self-perceived family wealth [β_d_ = 0.12; (95%CI = 0.04; 0.20)] exerted a standardized direct effect on BRIEF (Table 3). The standardized indirect effect of self-perceived family wealth and subjective health on NVS was [β_i_ = 0.01; (95%CI = 0.001; 0.03)] and [β_i_ = 0.02; (95%CI = 0.004; 0.05)].

In the “rural” subgroup, significant standardized direct effect of education [β_d_ = 0.12; (95%CI = 0.02; 0.24)], unmarried marital status [β_d_ = −0.13; (95%CI = −0.25;−0.01)], and social support [β_d_ = 0.19; (95%CI = 0.08; 0.30)] was observed on NVS. Education [0.17; (95%CI = 0.07; 0.27)], employment status [β_d_ = −0.18; (95%CI = −0.31;−0.03)], divorced marital status [β_d_ = −0.13; (95%CI = −0.24;−0.02)], and social support [β_d_ = 0.21; (95%CI = 0.10; 0.30)] had significant standardized direct effect on BRIEF (Table 3). Self-perceived family wealth [β_i_ = 0.03; (95%CI = 0.01; 0.06)] and subjective health [β_i_ = 0.04; (95%CI = 0.01; 0.09]) had indirect effect on NVS. Social support also mediated the effect of the association between self-perceived family wealth [β_i_ = 0.03; (95%CI = 0.01; 0.07)], subjective health [β_i_ = 0.04; (95%CI = 0.01; 0.08)] and BRIEF.

We also tested the hypothesis that the model which contains the two health literacy variables together was invariant across the respondent's permanent residence. The unconstrained configural model (CM) provided good fit to the data, with χ^2^ (*p*–value) = 0.139; CFI = 0.996; GFI = 0.994; and RMSEA = 0.016 (PCLOSE = 1.000). Model 1 (restricting all path coefficients to be equal) was compared against the configural model (which allowed all path coefficients to vary across groups), yielding χ^2^(df)_Model(1)_ = 126.812 (88) and Δχ^2^ (df) = 88.392 (58) with *p-*value = 0.006 and ΔCFI = 0.015. Model 2 (constrained only social support and education-related path coefficients to be equal) was also not invariant by type of residence (Δχ^2^ (df) = 22.554 (10), *p* < 0.013 and ΔCFI = 0.017) (Table 4). Differences in the path coefficients impact the stability of the model across permanent residence, reflecting inconsistent estimates of the direct and indirect relationships among the studied groups.

**Table 4:** Goodness-of-fit statistics for tests of invariance analyses in multigroups by geographical residence.

**Model description**	**Comparative model**	**χ^2^ (df)**	**Δχ^2^ (df)**	Statistical significance^*^	**CFI**	**ΔCFI**
Configural model (CM); no equality constraints imposed	-	38.42 (30)	-	-	0.996	-
Model(1); All path coefficients constrained equal	CM vs. Model(1)	126.812 (88)	88.392 (58)	***p*** **= 0.006**	0.981	0.015
Model(2); social support and education related path coefficients constrained equal	CM vs. Model(2)	60.973 (40)	22.554 (10)	***p*** **= 0.013**	0.979	0.017

*χ^2^(df), model chi-squared statistic (model degrees of freedom); Δχ^2^ (df), refers to difference in χ^2^ values between models (df refers to difference in number of degrees of freedom between models); CFI, comparative fit index of the model; ΔCFI, refers to difference in CFI values between models;*

* *chi-squared difference test. Significant differences are marked in bold*.

## Discussion

As per the first aim of our study, social support and educational attainment were shown to be the most important determinants of health literacy after adjusting for the effect of other SEP and demographic variables. Regarding the second aim, the magnitude of effect of social support and educational attainment was different between the three types of settlements, the strongest being in rural areas.

Education and social support were associated with both types of HL measurements but self-perceived family wealth was only related to self-evaluated HL (measured by BRIEF) while perceived health was only related to performance-based health literacy (measured by NVS). So determinants of performance-based and self-evaluated health literacy only partially overlapped in our study. Possible explanations for this difference can only be speculatory. One potential explanation may be the nature of the instruments: perceived health is an excellent measure of objective health status that is why it has been widely used in health interview surveys (31). NVS as a performance-based tool is similar to perceived health inasmuch as both can be considered objective ways of assessing the underlying construct. In contrast, BRIEF as a measure of HL and self-perceived family wealth are rather more subjective approximations of their underlying constructs. Another explanation may be the difference in measurement properties of the two tools. As for identifying inadequate HL, BRIEF demonstrated an AUROC curve of 0.79, while this was 0.88 for NVS (2, 21, 27). Furthermore, BRIEF contains items regarding the understanding of both written and verbal information, while NVS includes numeracy related items besides the understanding of written information. The two tools measure different aspects of health literacy therefore it is not unreasonable to assume that their determinants also differ.

Univariate analysis did not yield differences in the level of health literacy by type of permanent residence. This is in line with the result of the Hungarian eHealth literacy survey which similarly to ours did not find difference between urban and rural populations (32). However, path coefficients related to social support and education did not support cross-residential invariance meaning that geographical differences can be assumed in the determinants of health literacy. Potential explanations for this difference are probably manifold intriguing. One may be statistical: the simple fact that association (for instance in the case of education) was not proven in all strata does not necessarily mean lack of such an association. The statistical power of our study might not have been high enough to find it. The level of social support was highest in the rural strata, potentially the reason for the strong effect in that strata.

Our results are in line with the conclusion of the systematic review of Aljassim and Ostini (8) who found that differences in health literacy between urban and rural groups disappeared after controlling for SEP; that urban-rural differences mostly exist in developing countries, and in studies where HL was assessed from a specific (e.g., disease-related knowledge) point as opposed to a general point of view. This can be potentially explained by the observation that people from lower SEP tend to live or move to rural areas with lower costs of living which is supported by our data as well. Therefore, the association between rurality and health literacy should be considered an artifact if the analysis is not controlled for SEP.

The association between HL and health status was most frequently adjusted for social support (33–36) or HL as a mediator between social support and health was investigated (37), so comparisons with our results are limited. We found only one publication with a research question similar to ours and its results do not contradict ours: social capital-related factors were associated with knowledge about Alzheimer's disease in older Korean Americans after controlling for SEP variables (38).

### Strengths and Limitations

Our study is limited by its cross-sectional design unable to reveal causality, and by most of the analyzed variables being ordinal which should be taken into account when evaluating the results. In the critical evaluation of the results it should be mentioned, that the statistical analysis did not take into account all possible confounding factors (e.g., intelligence, genetic factors) that may have contributed to the weak standardized coefficients. We used two measures to assess HL and one to assess social support which is a limitation in light of the wide selection of available tools for the assessment of both. Other measurement tools could and should also be tested. However, HL assessment tools can be grouped into two broad categories such as performance-based and self-evaluated measures, and one of each was used in the present study which can improve the generalizability of our results.

Our research fills a gap in knowledge regarding the potential differences in HL of rural and urban populations in Europe, and also contributes to understand whether the relationship between health literacy and its determinants differs between rural and urban populations.

### Conclusion

Our study calls attention to the importance of type of permanent residence as a geographical proxy of factors impacting on health literacy. Social support seems to be a mediator of the effect of SEP on health literacy which could be taken into account when designing interventions to improve health literacy, especially in rural areas. Further studies would be needed especially in rural communities to see whether improvement of social support could be utilized in projects to increase the level of health literacy. Community action groups, community sessions or clubs could be organized where the attainment of specific health-related goals would require learning along with strengthening community relations. Another option could be the employment of mediators who can actively participate in the education of community members while also supporting them and helping to improve interactions between individuals and the health system.

These recommendations are in line with a previously published health literacy intervention model (39) according to which HL interventions should target—among others—the social context by activities which strengthen social support, empower individuals, and also involves workers of the health system.

## Data Availability Statement

The raw data supporting the conclusions of this article will be made available by the authors, without undue reservation.

## Ethics Statement

Ethical review and approval was not required for the study on human participants in accordance with the local legislation and institutional requirements. Written informed consent for participation was not required for this study in accordance with the national legislation and the institutional requirements.

## Author Contributions

ÉB contribution to the conception of the work, supervision of data collection, interpretation of data, and drafting the article. FV data analysis, interpretation of data, visualization, and drafting the article. GM interpretation of data and drafting the article. KK contribution to the conception of the work, supervision, and critical revision of the article. All authors have read and agreed to the published version of the manuscript.

## Funding

This work was supported by the GINOP-2.3.2-15-2016-00005 project, which is co-financed by the European Union under the European Regional Development Fund. The funders have had no influence on the study design, data collection and analysis, interpretation of the results, writing of the manuscript, or in the decision to submit it for publication.

## Conflict of Interest

The authors declare that the research was conducted in the absence of any commercial or financial relationships that could be construed as a potential conflict of interest.

## Publisher's Note

All claims expressed in this article are solely those of the authors and do not necessarily represent those of their affiliated organizations, or those of the publisher, the editors and the reviewers. Any product that may be evaluated in this article, or claim that may be made by its manufacturer, is not guaranteed or endorsed by the publisher.

## Abbreviations

BRIEF: Brief Health Literacy Screening Tool
CI: confidence interval
CFI: comparative fit index
CM: configural model
GFI: goodness-of-fit index
HL: health literacy
HLS-EU: European Health Literacy Survey
HLS-EU-Q47: European Health Literacy Survey Questionnaire 47
NVS: Newest Vital Sign
PCLOSE: p of close fit
RMSEA: root-mean-square error of approximation
SD: standard deviation
SEP: socioeconomic position
S-TOFHLA: Short-Test of Functional Health Literacy in Adults.
